# Injektionstherapie (GLP1-Rezeptor Agonisten und Insulin) bei Typ 2 Diabetes mellitus (Update 2023)

**DOI:** 10.1007/s00508-023-02171-x

**Published:** 2023-04-20

**Authors:** Monika Lechleitner, Michael Roden, Raimund Weitgasser, Bernhard Ludvik, Peter Fasching, Friedrich Hoppichler, Alexandra Kautzky-Willer, Guntram Schernthaner, Rudolf Prager, Susanne Kaser, T. C. Wascher, Ausschuss Leitlinien

**Affiliations:** 1 Avomed-Arbeitskreis für Vorsorgemedizin und Gesundheitsförderung in Tirol, Innsbruck, Österreich; 2 Klinik für Endokrinologie und Diabetologie, Medizinische Fakultät, Heinrich-Heine-Universität, Düsseldorf, Deutschland; 3 Institut für Klinische Diabetologie, Deutsches Diabetes-Zentrum (DDZ), Leibniz-Zentrum für Diabetesforschung, Düsseldorf, Deutschland; 4 Deutsches Zentrum für Diabetesforschung (DZD e. V.), München-Neuherberg, Deutschland; 5 Abteilung für Innere Medizin, Privatklinik Wehrle-Diakonissen, Salzburg, Österreich; 6 Universitätsklinik für Innere Medizin I, LKH Salzburg – Universitätsklinikum der Paracelsus Medizinischen Privatuniversität, Salzburg, Österreich; 7 Medizinische Abteilung mit Diabetologie, Endokrinologie und Nephrologie, Klinik Landstraße, Wien, Österreich; 8 Medizinische Abteilung für Endokrinologie, Rheumatologie und Akutgeriatrie, Wilhelminenspital der Stadt Wien, Wien, Österreich; 9 Abteilung für Innere Medizin, Krankenhaus der Barmherzigen Brüder Salzburg, Salzburg, Österreich; 10 Gender Medicine Unit, Klinische Abteilung für Endokrinologie und Stoffwechsel, Universitätsklinik für Innere Medizin III, Medizinische Universität Wien, Spitalgasse 23, 1090 Wien, Österreich; 11 Department of Internal Medicine II, Medizinische Universität Wien, Wien, Österreich; 12 Stoffwechselzentrum im Rudolfinerhaus, Rudolfinerhaus Privatklinik, Wien, Österreich; 13 Department für Innere Medizin 1, Medizinische Universität Innsbruck, Innsbruck, Österreich; 14 Medizinische Abteilung, Hanusch-Krankenhaus, Wien, Österreich

**Keywords:** Injektionstherapie bei Typ 2 Diabetes, Injection therapy and type 2 diabetes mellitus

## Abstract

Die vorliegende Leitlinie nimmt Bezug auf die Indikation und praktische Umsetzung der Injektionstherapie (GLP1-Rezeptor Agonisten und Insulin) bei Typ 2 Diabetes.

## Abstract (en)

The present article is a recommendation of the Austrian Diabetes Association for the practical use of injection therapy (GLP1-receptor agonists and insulin) in type 2 diabetes

## Indikationen zur Injektionstherapie

Eine Indikation zur Therapie mit GLP1-Rezeptor Agonisten (GLP1-RA) oder Insulin besteht, wenn durch diätetische Maßnahmen und orale Antidiabetika das individuelle Therapieziel in der Glukosekontrolle nicht erreicht wird. Falls möglich, wird vorrangig eine Therapieerweiterung mit einem **GLP1-RA** empfohlen [1, 2] und erst in einem weiteren Schritt die Insulingabe. Klinische Studien und Meta-Analysen beschreiben für GLP1-RA im Vergleich zu Insulin eine ähnliche oder größere Effektivität hinsichtlich der Verbesserung der Glukosekontrolle [3–5].

Unabhängig von der glykämischen Kontrolle besteht eine Indikation für eine GLP1-RA Therapie bei Patient:innen mit kardiovaskulärer oder renaler Vorerkrankung oder hohem kardiovaskulären Risiko, sofern keine SGLT‑2 Inhibitor Therapie vorliegt. Bei Vorliegen einer Kombinationstherapie aus Metformin und SGLT‑2 Inhibitoren bei kardiovaskulär/renal Vorerkrankten bzw. Patient:innen mit sehr hohem kardiovaskulären Risiko wird bei Nichterreichen des HbA1c Zielwertes der Beginn einer GLP1-RA Therapie mit nachgewiesenem kardiovaskulären Benefit empfohlen (Tab. 1 und 2).

**Table 1:** 

GLP-1-Rezeptor Agonist	Eliminationshalbwertszeit	Dosierungsintervalle	Administration
**KURZE WIRKDAUER**			
Exenatid (Byetta)	3,3–4,0 h	2 × täglich	Subcutane Injektion
Lixisenatid (Lyxumia)	2,6 h	1 × täglich	Subcutan Injektion
**INTERMEDIÄRE WIRKDAUER**			
Liraglutid (Victoza)	12,6–14,3 h	1 × täglich	Subcutane Injektion
Semaglutid (Rybelsus)	5,7–6,7 Tage	1 × täglich	Orale Gabe
**LANGE WIRKDAUER**			
Dulaglutid (Trulicity)	4,7–5,5 Tage	1 × wöchentlich	Subcutane Injektion
Semaglutid (Ozempic)	5,7–6,7 Tage	1 × wöchentlich	Subcutane Injektion

**Table 2:** 

	ELIXA (*n* = 6068)	LEADER (*n* = 9340)	SUSTAIN (*n* = 3297)	EXSCEL (*n* = 14.752)	REWIND (*n* = 9901)	PIONEER‑6 (*n* = 3183)
Intervention	Lixisenatid/Placebo	Liraglutid/Placebo	Semaglutid sc/Placebo	Exenatid QW/Placebo	Dulaglutid/Placebo	Semaglutid oral/Placebo
Einschlusskriterien	Typ 2 DM und Anamnese ACS (< 180 Tage)	Typ 2 DM und vorbekannte CV-Erkrankung, CKD, oder Herzinsuff mit > 50 Jahren oder CV-Risikoerhöhung im Alter > 60 Jahren	Typ 2 DM und vorbekannte CV-Erkrankung, Herzinsuffizienz, oder CKD im Alter > 50 Jahren oder CV-Risikoerhöhung im Alter > 60 Jahren	Typ 2 DM mit oder ohne vorbekannte CV-Erkrankung	Typ 2 DM und CV-Ereignis oder erhöhte Risikofaktoren	Typ 2 DM mit hohem CV-Risiko (Alter über 50 Jahre mit manifester CV-Erkrankung oder CKD, oder Alter > 60 Jahre mit CV-Risikofaktoren)
Alter (Jahre)/% Männer	60,3/69,3	64,3/64,3	64,6/60,7	62/62	66,2/53,7	66/68,4
Diabetesdauer (Jahre)	9,3	12,8	13,9	12	10,5	14,9
HbA1c (%) (Einschlusskriterien)	5,5–11	> 7,0	> 7,0	6,5–10,0	< 9,5	Keine
Mediane Beobachtungszeit (Jahre)	2,1	3,8	2,1	3,2	5,4	1,3
Metformin-Therapie (%)	66	76	73	77	81	77,4
HbA1c-Gruppen Unterschied am Ende der Therapie	−0,3	−0,4	−0,7 oder −1,0	−0,53	−0,61	−0,7
Primärer Endpunkt	4‑Punkt MACE 1,02	3‑Punkt MACE 0,87	3‑Punkt MACE 0,74	3‑Punkt MACE 0,91	3‑Punkt MACE 0,88	3‑Punkt MACE 0,79
CV-Mortalität	0,98 (0,78–1,22)	0,78 (0,66–0,93)	0,98 (0,65–1,48)	0,88 (0,76–1,02)	0,91 (0,78–1,06)	0,49 (0,27–0,92)
Myokardinfarkt	1,03 (0,87–1,22)	0,86 (0,73–1,00)	0,74 (0,51–1,08)	0,97 (0,85–1,10)	0,96 (0,79–1,15)	1,18 (0,73–1,90)
Schlaganfall	1,12 (0,79–1,58)	0,86 (0,71–1,06)	0,61 (0,38–0,99)	0,85 (0,70–1,03)	0,76 (0,61–0,95)	0,74 (0,35–1,57)
Hospitalisierung bei HI	0,96 (0,75–1,23)	0,87 (0,73–1,05)	1,11 (0,77–1,61)	0,94 (0,78–1,13)	0,93 (0,77–1,12)	0,86 (0,48–1,55)
Gesamtmortalität	0,94 (0,78–1,13)	0,85 (0,74–0,97)	1,05 (0,74–1,50)	0,86 (0,77–0,97)	0,90 (0,80–1,01)	0,51 (0,31–0,84)
Verschlechterung der Nephropathie	–	0,78 (0,67–0,92)	0,64 (0,46–0,88)	–	0,85 (0,77–0,93)	–

**Figure 1:**
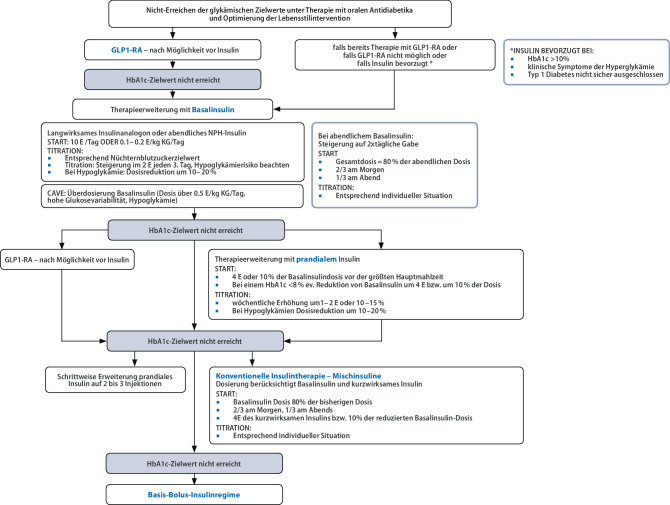

Das Sekundärversagen einer Therapie mit oralen Antidiabetika stellt in der klinischen Praxis die häufigste Indikation für die **Insulintherapie** bei Typ 2 Diabetes mellitus dar (Abb. 1). Auch Kontraindikationen gegenüber oralen Antidiabetika oder GLP1-RA, sowie schwere Allgemeinerkrankungen oder die perioperative Situation, können eine Insulintherapie vorübergehend oder dauerhaft erforderlich machen [1]. Eine Indikation zur Insulintherapie besteht auch bei hyperglykämischen Entgleisungen mit Glukosewerten über 300 mg/dL, insbesondere bei klinischen Symptomen (Polyurie, Polydipsie, Gewichtsreduktion) und/oder einem HbA_1c_-Wert über 10 % (85,79 mmol) [1].

Eine Therapieerweiterung durch eine **Kombination eines GLP1-RA mit Insulin** wird bei Nicht-Erreichen der glykämischen Zielwerte unter oraler Kombinationstherapie (2 oder 3 orale Antidiabetika) und einem HbA_1c_-Wert über 10 % bzw. 2 % über dem angestrebten Zielwert empfohlen [1, 2, 6].

Das Therapieregime sollte in regelmäßigen Intervallen (3–6 Monate) überprüft und an die Qualität der Blutzuckerkontrolle angepasst werden [1].

## GLP1-RA

GLP1-RA weisen bei einer effektiven Verbesserung der glykämischen Kontrolle ein niedriges Hypoglykämierisiko und vorteilhafte Gewichtseffekte auf [1, 7]. Die Wirkdauer bzw. das Dosierungsintervall für die in der Therapie des Typ 2 Diabetes mellitus zur Verfügung stehenden GLP1-RA sind in Tab. 1 dargestellt [7]. In einer Reihe von klinischen Studien konnten für GLP1-RA günstige kardiovaskuläre Effekte aufgezeigt werden (Tab. 2; [8]). (Siehe auch Medikamentöse Therapie des Typ 2 Diabetes mellitus).

### Initiale Injektionstherapie: GLP1-Rezeptor Agonist oder Insulin

Im ADA-EASD Consensus-Report 2019 wird der initialen Injektionstherapie den GLP‑1 RA gegenüber Insulin der Vorzug gegeben [6]. Die Argumente für diese Therapieempfehlung sind in der Tab. 3 zusammengefasst [2, 4]. Die glykämische Kontrolle ist mit GLP1-RA gleich gut oder sogar besser als mit Insulin, der Blutzucker wird zusätzlich auch postprandial gesenkt, während Basalinsuline (Glargin, Degludec) nur den Nüchternblutzucker senken. In klinischen Studien wurde unter GLP1-RA eine Gewichtsabnahme von 1,5–6 kg beobachtet, während unter einer Insulintherapie Gewichtszunahmen von 3–9 kg innerhalb des ersten Jahres nach Therapiebeginn beobachtet wurden [2]. Aufgrund des weitgehend fehlenden Risikos von Hypoglykämien sind im Gegensatz zur Insulintherapie häufig notwendige kostenintensive Blutzuckermessungen kaum notwendig. Im Gegensatz zur Insulintherapie fanden sich in zahlreichen Outcome-Studien mit GLP1-RA eindrucksvolle Senkungen der kardiovaskulären Komplikationen [7].

**Table 3:** 

	Insulin	GLP‑1-RA
Glykämische Kontrolle	Sehr effektiv	Sehr effektiv bei voller Dosis
Köpergewicht	Gewichtszunahme	Gewichtsabnahme
Hypoglykämierisiko	JA	NEIN, aber Zunahme des Risikos in Kombination mit Insulin und Insulinsekretagoga
Kardiovaskuläre Vorteile	Neutral	Vorteile bei Patienten mit manifesten kardiovaskulären Erkrankungen
Administration	1–4 mal tägliche Gabe	1–2 mal täglich und bei einigen Präparaten 1 mal wöchentlich
Monitoring	Glukosekontrolle ist essentiell	Blutzuckerselbstkontrolle vor allem wichtig bei Kombination mit Insulin und Insulinsekretagoga (Hypoglykämierisiko)
Nebenwirkungen	Lokalreaktionen an der Injektionsstelle sind selten	Gastrointestinale Beschwerden, innerhalb der Klassen unterschiedliches Risiko für Lokalreaktionen an der Injektionsstelle
Sicherheitsrisiken	Hypoglykämierisiko	Cholecystolithiasis assoziierte Ereignisse, aber keine Zunahme des lithogenen Pancreatitisrisikos

In rezent publizierten Studien konnte der Vorteil einer Therapieerweitung mit dem dualen Wirkstoff Tirzepatid, einer Kombination eines glucose-dependent insulinotropic Peptides (GIP) mit einem GLP1-RA, gegenüber eine Therpieerweiterung mit Insulin Degludec aufgezeigt werden. Bei Patient:innen mit Typ 2 Diabetes und Metformin-Vortherapie fand sich unter Tirzepatid eine stärkere Reduktion des HbA1c-Wertes, eine Reduktion des Körpergewichts und ein niedriges Hypoglykämierisiko [9]. Das Sicherheitsprofil von Tirzepatid entspricht jenem der GLP‑1 RA [10]. Die Europäische Arzneimittelbehörde hat im September 2022 Tirzepatid zur Therapie des Typ 2 Diabetes mellitus bei Erwachsenen zugelassen.

## Insuline

Zur Insulintherapie stehen kurzwirksame und langwirksame Insuline und Insulinanaloga, sowie Mischinsuline aus NPH-Insulin mit Normalinsulin bzw. kurzwirksamen Insulinanaloga zur Verfügung [1]. Als Basalinsulinanaloga der zweiten Generation werden die ultralangwirksamen Insuline Glargin U 300 und Degludec bezeichnet (siehe Abschnitt Typ 1 Diabetes) [11–13]. Ultrakurzwirksame Insulinanaloga sind Ultra Rapid Lispro [14] und Faster Aspart [15]. Als Nebenwirkungen der Insulintherapie müssen das erhöhte Hypoglykämierisiko, insbesondere bei der Verwendung kurzwirksamer Insuline, Spritzstellenveränderungen und das Potenzial zur Gewichtszunahme beachtet werden [1]. Ultra Rapid Lispro kann gelegentlich zu brennenden Missempfindungen an der Injektionsstelle führen, insbesondere bei der kontinuierlichen Verabreichung im Rahmen einer Pumpentherapie. Zu den Vorteilen der ultrakurzwirksamen Insulinanaloga zählen aufgrund des raschen Wirkeintritts und der kürzeren Wirkdauer die günstigere Wirkkinetik in Bezug auf die postprandialen Blutzuckerspitzen und ein niedrigeres Hypoglykämierisiko.

Die Entwicklung **langwirksamer Insulinanaloga** hatte zum Ziel, eine gegenüber NPH-Insulin flachere Wirkkurve und längere Wirkdauer zu erzielen. Langwirksame Insulinanaloga zeigten in klinischen Studien gegenüber NPH-Insulin eine Reduktion vor allem nächtlicher Hypoglykämien bei vergleichbarer Effektivität hinsichtlich der Erreichung der Blutzuckerzielwerte [16, 17]. Von Vorteil in der klinischen Praxis und Handhabung ist auch das Vorliegen der langwirksamen Insulinanaloga in Form einer klaren Lösung, während bei Applikation von NPH-Insulin eine vorausgehende Suspension des Insulins durch Schwenken erforderlich ist.

Als **ultralangwirksame Insulinanaloga** gelten Insulin **Glargin U 300** und Insulin **Degludec** [13]. Beide ultralangwirksamen Insuline stellen keine neuen molekulare Strukturen dar, sondern beruhen auf chemischen Modifikationen von Insulin Glargin U 100 und Insulin Detemir. Die Wirkdauer von Insulin Glargin U 300 beträgt über 30 h (Halbwertszeit 18–19 h), die Wirkdauer von Insulin Degludec rund 42 h (Halbwertszeit 25 h). Der Steady State wird bei täglicher Gabe bei beiden ultralangwirksamen Insulinen nach rund 4 Tagen erreicht [18].

Die lange Wirkdauer und flache Wirkkurve der ultralangwirksamen Insulinanaloga ermöglicht eine Reduktion der Injektionshäufigkeit des basalen Insulins – üblicherweise auf einmal täglich – und größere Flexibilität in der Wahl des Injektionszeitpunktes. In klinischen Studien wurde für die ultralangwirksamen Insuline insgesamt eine gegenüber Insulin Glargin U 100 geringere Hypoglykämierate und geringere Variabilität der Blutzuckerschwankungen beschrieben [19–22]. Für beide ultralangwirksamen Insulinpräparate liegen Studienreihen zum Einsatz bei Patient:innen mit Typ 1 und Typ 2 Diabetes mellitus vor.

Die Wirkeffektivität von **Insulin Glargin U 300** wurde in der EDITION-Studienreihe untersucht, die Teilstudien 1–3 inkludierten dabei Patient:innen mit Typ 2 Diabetes [21]. Als klinisch wichtiger Aspekt zeigt sich in einer Meta-Analyse das unter Insulin Glargin U 300 gegenüber Glargin 100 deutlich niedrigere Hypoglykämierisiko, vor allem für schwere Hypoglykämien [21].

Die Wirkeffektivität von **Insulin Degludec **wurde in der BEGIN- und in der DEVOTE-Studienreihe untersucht [22–24]. In der DEVOTE-Studie war bei einer Studienpopulation von kardiovaskulären Hochrisikopatient:innen die Inzidenz von schweren und nächtlichen Hypoglykämien unter Insulin Degludec gegenüber Insulin Glargin U 100 deutlich vermindert [23–25].

Kurzwirksame Insulinanaloga (Aspart, Lispro, Glulisin) und ultrakurzwirksame Insulinanaloga (Ultra Rapid Lispro, ultrakurzwirksames Insulin Aspart) weisen gegenüber Normalinsulin eine verbesserte Nachbildung des physiologische Insulinpeaks auf [1, 14, 15]. Aufgrund des raschen Wirkeintritts kann in der Regel auf einen Spritz-Essabstand verzichtet werden (siehe Leitlinie „Diagnostik und Therapie des Typ 1 Diabetes mellitus“).

### Formen der Insulintherapie

Für die Insulintherapie bei Patient:innen mit Typ 2 Diabetes stehen unterschiedliche Behandlungsvarianten zur Verfügung. Die Therapiewahl sollte Bezug auf die individuelle Situation der Patient:innen nehmen mit Berücksichtigung des Hypoglykämierisikos, der Gewichtseffekte und der Umsetzbarkeit im Alltag.

### Kombination von Insulin mit oralen Antidiabetika

Die **Basalinsulin-unterstützte orale Therapie (BOT)** mit einer Erweiterung der Therapie mit oralen Antidiabetika durch ein langwirksames Basalinsulin (NPH-Insulin, langwirksames Insulinanalogon, Insulinanalogon der zweiten Generation) gilt als einfache und in der Praxis erprobte Möglichkeit zur initialen Insulinisierung bei Nichterreichen des HbA_1c_-Zielwertes [1]. Als Startdosis werden 0,1 E/kg Körpergewicht bzw. 6–10 E Basalinsulin empfohlen, die Titration der Insulindosierung erfolgt anhand der Nüchternglukosewerte (Abb. 1).

Einzelstudien und Meta-Analysen konnten zeigen, dass die Kombination von Insulin mit oralen Antidiabetika bei Menschen mit Typ 2 Diabetes zu einer bis zu 40 %igen Einsparung des Insulinbedarfs gegenüber der alleinigen Insulintherapie führt [26, 27]. Zur Reduktion der Insulinresistenz und wegen der günstigen Gewichtseffekte wird ein Beibehalten von **Metformin** bei jeder Form der Insulintherapie bei Typ 2 Diabetes empfohlen [28]. Die Kombination eines Insulins mit einem **Sulfonylharnstoffderivat** ist mit einem erhöhten Hypoglykämierisikos assoziiert, die Kombination von Insulin mit **Pioglitazon** mit einer Neigung zu Flüssigkeitsretention und Ödementwicklung [1].

Günstige Gewichtseffekte wurden in klinischen Studien für die Kombination von Insulin mit **SGLT‑2 Inhibitoren** und Metformin beschrieben [29, 30]. Im Hinblick auf die vorteilhaften kardiovaskulären und renalen Effekte der SGLT-2-Hemmer sollte diese Substanzklasse auch im Rahmen der Insulintherapie bei Typ 2 Diabetes fortgeführt werden, sofern keine Kontraindikation besteht. In den klinischen Studien über die Sicherheit und Effektivität der SGLT2-Hemmer war ein Teil der Patient:innen, in der EMPA-REG-Studie 40 %, auch mit Insulin behandelt [31]. Als seltene Komplikation einer Therapie mit SGLT‑2 Inhibitoren, vor allem bei prädisponierenden Faktoren wie Insulinmangel und akuten Erkrankungen (insbesondere akuten Infekten), gilt die euglykämische Ketoazidose [32].

Auch für unterschiedliche **DPP‑4 Hemmer** liegen klinische Studien über die Effektivität und Sicherheit einer Kombination mit Basalinsulin vor [33].

Bei Nichterreichen der Glukosezielwerte unter Basalinsulin-unterstützer oraler Therapie wird eine Therapieerweiterung bzw. Therapieumstellung empfohlen (Abb. 1).

### Kombination von Insulin mit GLP1-RA

Für die Kombination eines langwirksamen Basalinsulins mit einem GLP1-RA konnte in klinischen Studien eine effektive und anhaltende Verbesserung der Glukosekontrolle aufgezeigt werden [1, 2, 34, 35]. Diese Therapieform stellt eine Alternative zu einer Erweiterung der Basalinsulingabe durch ein prandiales Insulin, zu einer Basis-Bolus-Insulintherapie bzw. einer konventionellen Insulintherapie mit Mischinsulinen dar. Vorteilhaft ist die Kombination eines Basalinsulins mit einem GLP1-RA hinsichtlich des Hypoglykämierisikos, sowie der günstigen Gewichtseffekte bei übergewichtigen und adipösen Patient:innen [36].

Für die Umsetzbarkeit einer Injektionstherapie bei Patient:innen, die Fremdhilfe benötigen, kann die Kombination eines einmal wöchentlich zu verabreichenden GLP1-RA mit einem langwirksamen, einmal täglich applizierbaren Basalinsulin vorteilhaft sein [1].

### Konventionelle Insulintherapie

Die konventionelle Insulintherapie ist durch eine verbindliche Vorgabe sowohl der Insulindosis als auch der Abfolge und Größe der Mahlzeiten charakterisiert. Bei dieser Therapieform werden Mischinsuline eingesetzt, deren Verabreichung meist 2‑ bis 3‑mal täglich erfolgt. Bei Typ 2 Diabetes beträgt die durchschnittliche Insulintagesdosis 0,5–1,0 E/kg Körpergewicht. Bei zweimal täglicher Gabe am Morgen und am Abend erfolgt die Aufteilung der Insulintagesdosis in einem Verhältnis von 2:1 bis 1:1. Mischinsuline von NPH-Insulin mit kurzwirksamen Insulinanaloga ermöglichen ein Weglassen des Spritz-Ess-Abstandes (siehe Leitlinie „Diagnostik und Therapie des Typ 1 Diabetes mellitus“). Titrationsschemata erleichtern die Anpassung der Insulindosis an die aktuellen Blutzuckerwerte [1, 37, 38]. Die konventionelle Insulintherapie findet aufgrund der geringen Flexibilität vor allem bei Patient:innen mit regelmäßigem Tagesablauf Anwendung.

Die Therapie des Mischinsulins aus 70 % Insulin Degludec und 30 % Insulin Aspart erfolgt als 1‑ oder 2‑mal tägliche Verabreichung zur Hauptmahlzeit. Klinische Studien weisen auf eine im Vergleich zur Fixmischung aus 70 % NPH-Insulin und 30 % Insulin Aspart ähnliche Wirkeffektivität bei geringerem Hypoglykämierisiko hin [39].

### Getrennte Verabreichung von Basal- und Bolusinsulin

Eine Therapie mit getrennter Verabreichung von Basal- und Bolusinsulin kommt vor allem bei Patient:innen mit flexiblem Tagesablauf und Bereitschaft zu häufigeren Glukosekontrollen (Glukosesensormessungen) zum Einsatz. Eine Zwischenvariante stellt die Erweiterung der Basalinsulintherapie mit zunächst einmaliger Gabe eines kurzwirksamen Insulins/Insulinanalogons zur Hauptmahlzeit dar („BOT-plus-Therapievariante“) dar (Abb. 1; [40]). Sulfonylharnstoffderivate sollten wegen des erhöhten Hypoglykämierisikos abgesetzt werden [1].

In der Folge kann die Insulintherapie dann schrittweise durch bedarfsgerechte Zugabe eines Bolusinsulin zu den weiteren Mahlzeiten intensiviert werden (Basis-Bolus-Therapie).

Im Rahmen der funktionellen Insulintherapie erfolgt die getrennte Substitution des basalen und prandialen Insulinbedarfs mit einer Anpassung der Insulindosierung durch die Patient:innen selbst (siehe Leitlinie „Diagnostik und Therapie des Typ 1 Diabetes mellitus“). Zur erfolgreichen Umsetzung der funktionellen Insulintherapie sind entsprechende Schulungsmaßnahmen sowie die Fähigkeit und Bereitschaft zur Übernahme der Entscheidungskompetenz durch die Patient:innen besonders wichtig [41].

Die Insulinpumpentherapie und hybrid Closed Loop Therapie stellen Varianten der funktionellen Insulintherapie dar (siehe Abschnitt Insulinpumpentherapie), die bei Menschen mit Typ 2 Diabetes nur bei besonderen Gegebenheiten in Erwägung gezogen werden sollte (ausgeprägtes Dawn Phänomen, Schwangerschaft – siehe Leitlinien „Technische Diabetestherapie“) [42].

## Interessenkonflikt

P. Fasching: Vortragshonorare und Honorare für Consulting bzw. Fortbildungsförderung (Kongressunterstützung): Astra Zeneca, Boehringer Ingelheim, Eli Lilly, Novo Nordisk, Sanofi, Viatris. Teilnahme an Klinischen Studien: Boehringer Ingelheim, Eli Lilly, Novo Nordisk, Sanofi. F. Hoppichler hat von folgenden Unternehmen, die auch fördernde Mitglieder der ÖDG sind, Forschungsunterstützungen und/oder Honorare erhalten: Astra Zeneca, Boehringer Ingelheim, Eli Lilly, Novartis, Novo Nordisk, Sanofi Aventis. S. Kaser hat von folgenden Unternehmen, die zT auch fördernde Mitglieder der ÖDG sind, Forschungsunterstützungen und/oder Honorare erhalten: AstraZeneca, Boehringer Ingelheim, Eli Lilly, Merck Sharp & Dohme, Novartis, Novo Nordisk, Viatris. A. Kautzky-Willer hat von folgenden Unternehmen, die auch fördernde Mitglieder der ÖDG sind, Forschungsunterstützungen und/oder Honorare erhalten: AstraZeneca, Amgen, Boehringer Ingelheim, Eli Lilly, Merck Sharp & Dohme, Novartis, NovoNordisk, sanofi-aventis. M. Lechleitner: Vortragstätigkeit und Teilnahme an Advisory Boards: Boehringer Ingelheim, Daiichi Sankyo Austria, Sanofi, Novo Nordisk. B. Ludvik erhielt Honorare für Vorträge, die Teilnahme an Advisory Boards und Forschungsunterstützung von: Amgen, Astra Zeneca, Bayer, Boehringer Ingelheim, Daiichi Sankyo, Eli Lilly, MSD, Novartis, Novo Nordisk, Sanofi. R. Prager: klinische Studien Novo Nordisk. M. Roden hat von folgenden Unternehmen Forschungsunterstützungen und/oder Honorare erhalten: Allergan, Astra Zeneca, Boehringer-Ingelheim, Bristol-Myers Squibb, Eli Lilly, Fishawack Group, Gilead, Genentech, Intercept Pharma, Inventiva, Merck, Novartis, Novo Nordisk, Nutricia/Danone, Pfizer, Poxel, Prosciento, sanofi-aventis, Target RWE. G. Schernthaner: Keine Interessenskonflikte. T. C. Wascher hat von folgenden Unternehmen, die teils auch fördernde Mitglieder der ÖDG sind, Forschungsunterstützungen und/oder Honorare erhalten: Abbott, Amgen, Astra Zeneca, Boehringer-Ingelheim, Daiichi-Sankyo, Eli Lilly, MSD, Novartis, Novo Nordisk, Roche, Sanofi-Aventis. R. Weitgasser hat von folgenden Unternehmen, die teils auch fördernde Mitglieder der ÖDG sind, Forschungsunterstützungen und/oder Honorare erhalten: Abbott, Amgen, AstraZeneca, Boehringer-Ingelheim, Daiichi-Sankyo, Eli Lilly, Merck Sharp & Dohme, Novo Nordisk, Roche, Sanofi.
